# Commentary: The pharmacogenomic landscape of an Indigenous Australian population

**DOI:** 10.3389/fphar.2024.1373056

**Published:** 2024-05-15

**Authors:** Cassandra White, Christine Paul, Rodney J. Scott, Stephen Ackland

**Affiliations:** ^1^ Hunter Medical Research Institute, The University of Newcastle, New Lambton, NSW, Australia; ^2^ The University of Newcastle, Callaghan, NSW, Australia; ^3^ Maitland Hospital, Metford, NSW, Australia; ^4^ Hunter Medical Research Institute, New Lambton, NSW, Australia; ^5^ Lake Macquarie Private Hospital, Gateshead, NSW, Australia

**Keywords:** fluoropyrimidine, DPYD gene, UGT1A1 gene, ethnicity, pharmacogenenomics and personalised medicine

We write to commend Samarasinghe and colleagues on their collaborative and progressive research in Indigenous Australian health and to acknowledge the important clinical implications of some of their findings. We address the significance of this article as it pertains to the safe prescription of chemotherapies for solid organ cancers.

Samarasinghe et al. conducted an analysis utilising whole genome sequencing on blood samples from 473 Tiwi Indigenous people looking specifically at the allele frequency of Very Important Pharmacogenes (VIP), as determined by PharmKGB (pharmkgb.org) (Samarasinghe et al., 2023). Our specific interest relates to the dihydropyrimidine dehydrogenase (*DPYD*) and uridine diphosphate-glucuronosyltransferase isoform 1A1 (*UGT1A1*) gene data.

Typical *DPYD* variants with clinical significance described within Caucasian populations include 4 main variants; c.1905 + 1G>A, (*2A, rs3918290), c.1679T>G (*13, rs55886062), c.2846A>T (rs67376798) and c.1236G>A/HapB3 (rs56038477) (Amstutz et al., 2018). Allele frequencies for these variants including Tiwi data are summarised in Table 1. Caucasian individuals who carry these variants typically have a deficiency in the dihydropyrimidine dehydrogenase (DPD) enzyme, the critical enzyme involved in the metabolism of fluoropyrimidine chemotherapies 5-fluorouracil and capecitabine, and can develop significant and life-threatening toxicity on exposure to these medications (Froehlich et al., 2015). Interestingly, of the 160+ known *DPYD* variants only seven distinct *DPYD* variants were identified within this Tiwi cohort; six variants deemed ‘normal metabolisers’ and c.1236G>A which is known to be clinically significant (Amstutz et al., 2018; Varughese et al., 2020; Samarasinghe et al., 2023). It is very significant that none of the 473 individuals screened carried c.1905 + 1G>A, c.1679T>G, c.2846A>T variants. Only one person was identified to carry c.1236G>A (HapB) in compound heterozygosity with ‘normal metaboliser’ variant c.1627A>G (*5, rs1801159). Fifty-seven individuals were found to be heterozygote for c.1627A>G with an additional 3 homozygote carriers. Nine individuals were found to carry c.2194G>A (*6, rs1801160). Both *DPYD* c.1627A>G and c.2194G>A are described as ‘non-significant’ variants in international guidelines, though there is conflicting data from non-Caucasian populations describing DPD deficiency and FP toxicity in carriers (Zhang et al., 2007; Offer et al., 2013; Patil et al., 2016; Amstutz et al., 2018; Naushad et al., 2020; White et al., 2021). Both these variants are found in higher frequency in the Tiwi population than other ethnic groups (Table 1) and so genotype/phenotype correlation should be performed to establish whether Tiwi carriers are truly ‘normal metabolisers’. We acknowledge that the small population size likely contributes to the high allele frequencies. We also note that a small sample size may account for the absence of rare variants such as c.1679T>G.

**TABLE 1:** DPYD allele frequency.

	DPYD variant allele frequency						
Population	c.1236G>A rs56038477	c.1627A>G rs1801159	c.2194G>A rs1801160	c.1679T>G rs55886062	c.2846A>G rs67376798	c.577G>A rs115232898	c.1905 + 1G>A rs3918290
Tiwi	0.001	0.076	0.11	0.000	0.000	0.000	0.000
Admixed American	0.005	0.0002^a^	0.032	0.000	0.002	0.001	0.001
African/African American	0.003	0.0001^a^	0.025	0.0001^a^	0.001	0.021	0.001
Amish	0.029	0.000	0.018	0.000	0.000	0.000	0.000
Ashkenazi Jewish	0.006	0.000	0.108	0.000	0.000	0.000	0.006
East Asian	0.000	0.000	0.019	0.000	0.000	0.000	0.000
European (Finnish)	0.013	0.000	0.023	0.000	0.000	0.000	0.023
European (non-Finnish)	0.022	0.0005^a^	0.045	0.0008^a^	0.006	0.000	0.005
Middle Eastern	0.013	0.000	0.124	0.000	0.000	0.001	0.003
Remaining	0.016	0.0002^a^	0.056	0.0006^a^	0.004	0.002	0.005
South Asian	0.017	0.000	0.096	0.000	0.001	0.000	0.004
Total	0.019	0.0001	0.047	0.0007^a^	0.005	0.001	0.005

Tiwi data from Samarasinghe et al. (2023). Population data from gnomAD v4.0.0 (gnomad.broadinstitute.org). Rounded to 3 decimal places.

^a^ Rounded to 4 decimal places.

*UGT1A1* was also sequenced by Samarasinghe *et al* in relation to the metabolism of antiretroviral Atazanavir (Samarasinghe et al., 2023). This gene holds clinical significance in the administration of irinotecan chemotherapy (Iyer et al., 1998). *UGT1A1* is the critical enzyme of irinotecan metabolism, and all heterozygote carriers within this cohort are ‘intermediate metabolisers’ according to pharmacogenetics guidelines for irinotecan prescribing (Hulshof et al., 2022). Twenty-five heterozygote carriers of *6 and 18 heterozygous carriers of *28 (in compound with *80) were identified (Varughese et al., 2020; Hulshof et al., 2022; Samarasinghe et al., 2023). One homozygote of 28* (+80*) was identified, inferring ‘poor metabolism’ of irinotecan. Another significant variant, *37, was not identified within this cohort, and is typically only identified in African ethnic communities. Allele frequencies for these variants, including Tiwi data are described in Table 2. The clinical significance of *80 alone without compound of *28 or *37 is uncertain and it is not included as a clinically significant variant in current prescribing guidelines (Hulshof et al., 2022).

**TABLE 2:** UGT1A1 allele frequency.

	UGT1A1 variant allele frequency			
Population	*6 rs4148323	*28 rs8175347	*37 rs8175347	*80 rs887829
Tiwi	0.054	0.022	0.000	0.022
African American	0.004	0.373	0.057	0.450
Asian	0.146	0.148	0.000	Not reported
European	0.008	0.317	0.001	0.314

Tiwi data from Samarasinghe et al. (2023). Population data from Varughese et al. (2020).

These data highlight the importance of conducting genomic sequencing in ethnically diverse populations to better understand VIP allele frequencies and improve pharmacogenomic (PGx) prescribing universally, not just for Caucasian communities. Due to the potential for ethnicity-based differences in gene interactions, additional effort should be made to determine the phenotype of individuals found to harbour clinically significant variants, and phenotyping could extend to all *DPYD* variants identified within the Tiwi community, as ‘normal metaboliser’ variants may in fact show different expression in this and other Indigenous populations. Larger population sampling within ethnic minority populations would help to establish the frequency of very rare variants and allow more rigorous extrapolation of polymorphism frequencies.

Fluoropyrimidines and irinotecan chemotherapies are prescribed for over 16,000 Australians per year for the treatment of solid organ malignancies (Australian Institute of Health and Welfare A, 2019). In various countries across Europe patients are genotyped prior to receiving fluoropyrimidine chemotherapies as standard of care. Patients found to hold certain *DPYD* variants have chemotherapy doses adjusted prior to exposure to reduce the likelihood of severe toxicity (Deenen et al., 2016; Henricks et al., 2018). This is not currently mandated in Australia, despite recognition of its importance in both international and national prescribing guidelines (Argiles et al., 2020; White et al., 2022; NSW, 2023). There is increasing data to show that differing ethnic populations may not express the same phenotypically significant *DPYD* variants that are important in European Caucasian communities and may carry alternate variants that have clinical significance (White et al., 2021). It is extremely important that as Australia moves toward developing PGx screening that we consider our ethnically diverse community and invest effort in determining genomic differences that need to be included in our screening modalities, rather than modelling our approach directly from European data.

Not enough is known about the clinical significance of the variants identified in the Tiwi community and in fact in many other non-European ethnicities. Further research is required before dose-personalisation of potentially life-threatening drugs can be developed. Understanding the genotype/phenotype interplay for variants determined in non-Caucasian communities will help to guide dose-personalisation to allow for safe administration of necessary chemotherapeutic agents such as fluoropyrimidines and irinotecan while reducing the risk of severe chemotherapy induced toxicity and even treatment related death. PGx gene panels can be tailored to the reflect *DPYD* genetic expression within local jurisdictions for national screening efforts (Suarez-Kurtz et al., 2023). Indigenous communities throughout Australia are as diverse in geographical location as they are in cultural practice and collaborative effort to develop culturally sensitive and inclusive genetic screening to determine important variants should be sought in an effort to improve safe prescribing for all Australian cancer patients.

We support the continuation and expansion of collaborative research such as this, in conjunction with Indigenous communities, to explore and enrich our understanding of the genomic landscape of our diverse ethnic populations. This will help to tailor health interventions such as pharmacogenomic screening programmes like prospective *DPYD/UGT1A1* genotyping that will be meaningful for all ethnicities.
